# Activity Deprivation Induces Neuronal Cell Death: Mediation by Tissue-Type Plasminogen Activator

**DOI:** 10.1371/journal.pone.0025919

**Published:** 2011-10-06

**Authors:** Eldi Schonfeld-Dado, Menahem Segal

**Affiliations:** Department of Neurobiology, The Weizmann Institute of Science, Rehovot, Israel; University of Cincinnatti, United States of America

## Abstract

Spontaneous activity is an essential attribute of neuronal networks and plays a critical role in their development and maintenance. Upon blockade of activity with tetrodotoxin (TTX), neurons degenerate slowly and die in a manner resembling neurodegenerative diseases-induced neuronal cell death. The molecular cascade leading to this type of slow cell death is not entirely clear. Primary post-natal cortical neurons were exposed to TTX for up to two weeks, followed by molecular, biochemical and immunefluorescence analysis. The expression of the neuronal marker, neuron specific enolase (NSE), was down-regulated, as expected, but surprisingly, there was a concomitant and striking elevation in expression of tissue-type plasminogen activator (tPA). Immunofluorescence analysis indicated that tPA was highly elevated inside affected neurons. Transfection of an endogenous tPA inhibitor, plasminogen activator inhibitor-1 (PAI-1), protected the TTX-exposed neurons from dying. These results indicate that tPA is a pivotal player in slowly progressing activity deprivation-induced neurodegeneration.

## Introduction

Spontaneous ongoing network activity plays a critical role in neuronal development and survival, and regulates neuronal functionality in many neural systems [1]–[3]. It is manifested in the form of synchronized action potential discharges by neurons comprising a functional network, both in-vivo and in-vitro. Suppression of spontaneous ongoing network activity in culture using the voltage-gated sodium channel blocker TTX, can have fatal consequences to the exposed network which die following a prolonged period of activity deprivation [4]–[6]. This protracted cell death is caused by a gradual activation of cell death cascade and is accompanied by an increase in miniature excitatory post-synaptic currents (mEPSC) [4], proposed recently to be mediated by glial tumor necrosis factor (TNF) [7], another acknowledged regulator of cell death. Neuronal death is a key determinant in neurodegenerative diseases, and as yet there is little understanding of the molecular cascade leading to slow cell death, hence it is a major focus of attention in recent years.

Neuronal activity is responsible for the induction of key neuronal genes, known as activity-regulated genes (e.g. BDNF) [8]. One of them is the serine protease tPA, the principle enzyme responsible for converting plasminogen into its active form, plasmin. tPA mediates extracellular matrix degradation (and is therefore the only approved drug for acute ischemic stroke) [9] and affects neuronal interactions, but is also implicated in neuronal death cascades [10]–[13]. The aim of the present study was to further explore the mechanistic aspects that govern activity deprivation-induced neuronal cell death. We found that tPA is up-regulated in the chronically silenced neurons at both the mRNA and the protein levels, and that it regulates TTX induced neurotoxicity. We therefore propose tPA as a crucial mediator of the toxic effects exerted by neuronal activity deprivation.

## Results

**Figure 1:**
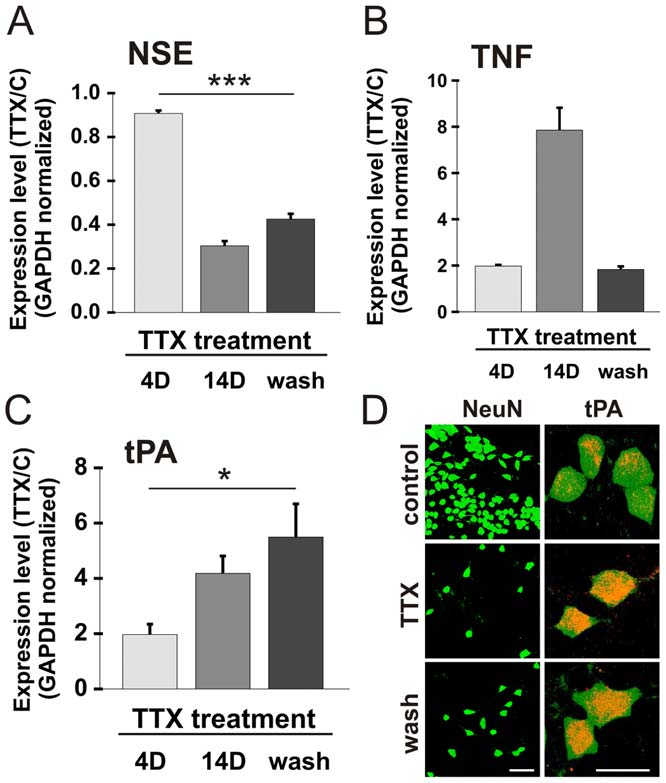
NSE is down-regulated and tPA is up-regulated during and following TTX application. (a–c) Real-time PCR analysis showing the change in the mean mRNA expression level of the neuronal marker NSE, TNF and tPA after short (4D) and long (14D) term TTX treatment and following 4 days of TTX treatment after which it was washed out and the cells were left to grow in regular medium for 10 more days (wash condition), relative to control (means±SEM; * = P<0.05; *** = P<0.001). (d) Immunoflorescence images of control, long term TTX-treated and TTX-washed cultures, stained for the neuronal marker NeuN (green - left panel, bar = 50 µm) or double-labeled for NeuN and for tPA (red - right panel, bar = 20 µm), showing the elevation in tPA protein levels among the treated neurons and its cellular localization.

We have previously shown that chronic blockade of activity by exposure to TTX results in a slow and progressive degeneration of the silenced neurons [5], [6]. This is expressed as a massive reduction in neuron number, and in a parallel reduction in the mRNA expression level of neuron specific enolase (NSE) (14D, Fig. 1A). We have also demonstrated the irreversibility of the death process, which starts after 4 days of exposure to TTX and proceeds even after TTX removal (designated the ‘wash’ period), through which NSE continues to decrease (Fig. 1A, n = 5 independent experiments, p = 2×10−9 - one way ANOVA, p = 0.97 - Shapiro-Wilk normality test). In order to gain additional mechanistic insights on cell death, we utilized real-time PCR analysis to examine the involvement of key genes in the process. One of the factors associated with the death process is tumor necrosis factor (TNF), which was indeed highly elevated during TTX treatment (8-fold, Fig. 1B), supporting previous suggestions [7]. However, TNF did not remain elevated upon TTX removal, indicating that it may not be involved in the death process, which is triggered by but no longer dependent on continuous TTX presence. In sharp contrast, tPA, maintained its high expression level also when the cells were dying after removal of TTX (4-fold and 5-fold, respectively, Fig. 1C, n = 5 independent experiments, p<0.05 - one way ANOVA, p = 0.52 - Shapiro-Wilk normality test), indicating that it may be involved in neuronal death caused by activity deprivation (Fig. 1A). This elevation was detected in tPA protein levels as well, as demonstrated by immunefluorescence staining (Fig. 1D), correlating neuronal population loss with the induction of tPA expression in individual neurons, and its concentration inside the nucleus of the dying TTX-treated or -washed cell, in contrast to its somatic localization in control neurons.

**Figure 2:**
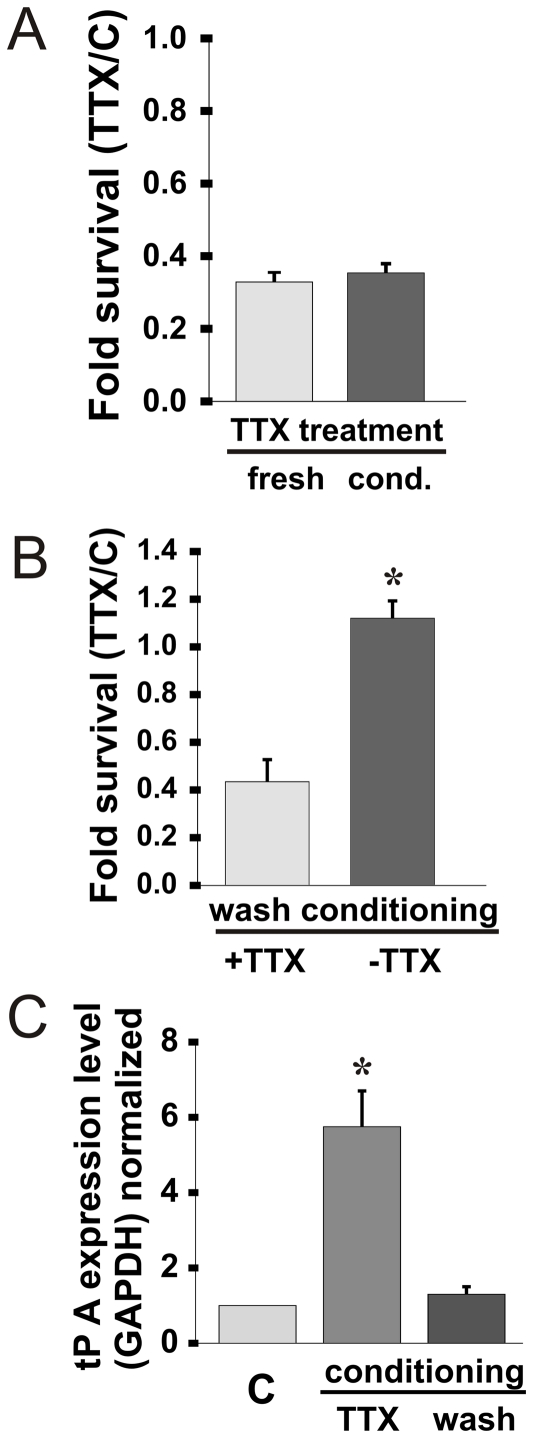
The effect of TTX and conditioned medium on the cell survival and tPA expression level in neuronal cultures. (a) Mean cell survival rates of primary neuronal cultures following the application of 3 days TTX-conditioned medium or fresh TTX for 7 days. (b) Cell survival rates of primary neuronal cultures following the application of conditioned medium of cells that were treated for 4 days with TTX (‘wash conditioning’) and left for 3 more days in fresh medium, with or without the addition of fresh TTX, for 7 days (c) The change in tPA mRNA expression level in primary neuronal cultures following a week of incubation in TTX- or wash-conditioned medium (means±SEM; * P<0.05).

To determine if indeed tPA operates through an intracellular route rather than the more prevalent extracellular arena, we tested for the presence of secreted toxic factors in both TTX-conditioned medium and in the medium conditioned by cells from which TTX was removed (‘wash conditioning’). Naïve cells were exposed to either fresh TTX, TTX-conditioned or wash-conditioned medium, and cell survival rates and tPA mRNA expression level were assessed (Fig. 2). While TTX-conditioned medium was as potent as medium containing fresh TTX in inducing neuronal cell death (Fig. 2A), wash-conditioned medium had no effect on cell viability unless TTX was added, in which case it induced death in 60% of the neurons (Fig. 2B, n = 3 independent experiments, p<0.03 - t-test, p = 0.83 - Shapiro-Wilk normality test). Under these conditions, tPA's mRNA level was elevated by roughly 6 fold in cells exposed to TTX-conditioned but not wash-conditioned medium (Fig. 2A, n = 3 independent experiments, p<0.04 - t-test, p = 0.83 - Shapiro-Wilk normality test).

**Figure 3:**
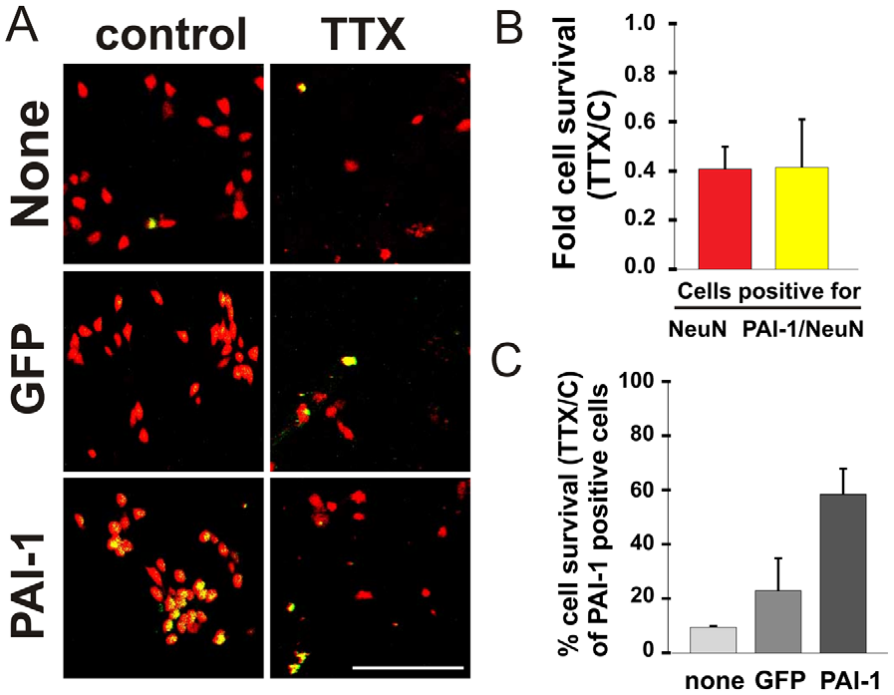
Intrinsic blockade of tPA protects neurons from TTX-mediated neuronal cell death. (a) Representative images of control and TTX-treated neuronal cultures stained for the neuronal marker NeuN (red), and for the tPA inhibitor PAI-1 (green), transfected with either GFP, PAI-1 or non-transfected controls (bar = 100 µm). (b) Control and TTX-treated neurons were stained for NeuN and for PAI-1. Cell survival rates of the NeuN-positive cells and of the PAI-1-positive cells out of the NeuN-positive population were estimated. (c) Cell survival rates of double-labeled cells of the three groups - Non-transfected, GFP and PAI-1, demonstrating the protection exerted by the over-expression of PAI-1.

If indeed tPA has a critical role in the process of neuronal cell death, then interfering with its action should reduce it. Neurons were transfected with the endogenous inhibitor of tPA, PAI-1 (a gift of Rene Bernards, The Netherlands), which resulted in an elevation in cell survival rates compared to cells transfected with GFP or non-transfected cells (Fig. 3A&C).This protective effect, observed in other model systems as well [14], was due to the induced over-expression of PAI-1 as TTX by itself did not cause any elevation in endogenous PAI-1 protein levels (as might be assumed to be the case if any internal survival pathway was activated, Fig. 3B), and indeed the enrichment in PAI-1-expressing neurons was due to the transfection as appeared in the immunefluorescence analysis (Fig. 3A).

## Discussion

The present results support a role for tPA in mediating activity deprivation-induced neuronal cell death. tPA's role in neuronal survival and death paradigms is quite diverse and controversial - there are many reports supporting its pro-survival effect on neurons both in-vivo and in-vitro [15]–[18], but other studies have demonstrated its toxic effect or the protective effect of its endogenous inhibitor PAI-1 when secreted from astrocytes [19]–[21]. Our study reinforces the proposed involvement of tPA in neuronal death cascades, and also provides evidence for neuronal-originated PAI-1 (rather than glial) being beneficial in counteracting tPA toxic effects.

tPA mRNA expression levels were found to be elevated throughout TTX treatment in a gradual manner, corresponding to neuronal demise (Fig. 1A&C). This was not different from other genes tested, such as TNF which is an important mediator of neuronal apoptosis (Fig. 1B), but unlike these other genes, tPA remained highly elevated during the recovery period in which cells were dying independently of TTX presence. Assuming that neuronal death mechanisms are the same for both the chronic and the ‘wash’ phases, it is reasonable to assume that tPA has a major role in the death process in both conditions. The elevation in the mRNA level was accompanied by a corresponding elevation in the intracellular and intranuclear tPA protein levels in the treated neurons (chronically and washed, Fig. 1D) compared to control cells, suggesting that in our case, tPA may play an intracellular role in the death cascade. Another reinforcement for the suggestion that tPA may not mediate the death process in an extracellular manner is in the observation that secreted factors are playing no role in this cell death process as demonstrated in Fig. 2, showing that TTX-conditioned medium (containing TTX) was as potent as freshly-added TTX in eliciting cell death, and that the conditioned medium of cells in their recovery period is not toxic at all to naïve cells (Fig. 2A&B). Similar to the induction of tPA by chronic TTX treatment, there was a linear relation between tPA induction and the severity of the cell death exerted by the different types of conditioned media (Fig. 2C). If tPA is to play a crucial role in this death process, then blocking its activity would result in a protective effect on the dying neurons. To test this possibility we utilized the endogenous inhibitor of tPA in the cell, PAI-1, which is implicated by itself as a neuronal protecting agent. PAI-1 over-expression resulted in an increase in cell survival rates among TTX-treated neurons relative to cell transfected with a GFP or non transfected at all (Fig. 3). As before, the transfection experiments reinforce the assumption that tPA acts intracellularly, since application of the recombinant PAI-1 protein to the growing medium of the cells was shown not to be effective at all in elevating cell survival rates, unlike the case of the expression plasmid (unpublished data).

One of the reasons that neurodegenerative diseases are of a major concern is the fact that they are terminal and cureless as they are often diagnosed too late for medical intervention. For this reason preventive medicine which will engage in the early, pre-symptomatic stages of the disease should be developed - by detecting suspicious signs in specific neuronal populations (for example, elevation in tPA protein which occurs already after 4 days of treatment, Fig. 1C) we could try and treat them locally (for example by attenuating tPA activity, Fig. 3C) and prevent their demise and the collateral damage accompanied to it. We therefore believe that our study places tPA in a promising position as a future therapeutic target for neuroprotection.

## Materials and Methods

### Cultures

Wistar rat pups were decapitated on postnatal day 3 (P3) and their brains removed and placed in a chilled (4°C), oxygenated Leibovitz L15 medium (Biological Industries, Beit Haemek, Israel) enriched with 0.6% glucose and Gentamicin (20 µg/ml; Sigma, St. Louis, MO). Bilateral cortical tissue was mechanically dissociated and plated on 12-mm glass coverslips at 4×10^5^ cells per well in a 24-wells plate, or on 35 mm culture dishes at 1.5 to 2×10^6^ cells. The plating medium consisted of 5% heat-inactivated horse serum (HS) and 5% fetal calf serum and was prepared in MEM (minimal essential medium)-Earl salts (Biological Industries, Beit Haemek, Israel) enriched with 0.6% glucose, Gentamicin, and 2 mM glutamax. Cells were left to grow in the incubator at 37°C, 5% CO_2_ for 4 days, at which time the medium was changed to 10% HS in enriched MEM, plus a mixture of 5′-fluoro-2-deoxyuridine/uridine (Sigma, 20 µg and 50 µg/ml, respectively). The medium is changed 4 days later to 10% HS in enriched MEM. TTX (at 1 µM, Alomone labs, Jerusalem, Israel) was added to the growth medium at 4 days in culture for up to 14 days of incubation period.

### Transfection

A lipofectamine 2000™ (Invitrogen, Carlsbad, CA, USA) mix was prepared at 1 µl/well with 50 µL/well optimem™ (Invitrogen, Carlsbad, CA, USA) and incubated for 5 min at room temperature. This was mixed with 1.5 µg/well total DNA in 50 µl/well optimem™ and incubated for 15 min at room temperature. The mix was then added to the cells and allowed to rest for 4–6 h until medium replacement. In most cases, at least several neurons were transfected. Co-transfection efficiency for several plasmids using this method was nearly 100%.

### Immunocytochemistry

Cultures were washed briefly with standard extracellular solution (129 mM NaCl, 4 mM KCl, 10.5 mM glucose and 10 mM and HEPES, pH = 7.4), fixed in 4% paraformaldehyde with 4% sucrose for 20 min, and washed with phosphate-buffered saline (PBS). For fluorescence staining cells were blocked for 1 h in 10% GS with 0.1% triton X-100 and incubated overnight at 4°C with mouse αNeuN (chemicon, Massachusetts, USA; 1∶1000), rabbit αtPA (1∶400, Santa Cruz, California, USA) or rabbit αPAI-1 (1∶500, Santa Cruz, California, USA) followed by 1 h secondary antibody labeling (Alexa 488-labeled and Alexa 546-labeled goat anti-mouse - Molecular Probes, Eugene, OR; 1∶200). Cover slips were washed again, transferred onto glass slides and mounted for visualization with anti-fading mounting medium. Confocal image stacks were recorded using a Zeiss LSM 510 laser scanning microscope, a Zeiss 40× and 100× oil immersion objective. About 4 fields are taken per 12 mm coverslip for statistical analysis, at each case.

### Quantitative reverse transcriptase (RT) Real Time-PCR analysis

Total cellular RNA was isolated using Qiagen RNeasy mini kit according to manufacture's instructions. Reverse transcription was performed with oligo dT primers and the RevertAid RT enzyme (Fementas) at 42°C for 60 min. The RT product from 500 ng of total RNA was applied to each 20-µL PCR reaction mixture containing 1× Absolute Blue Cyber Green PCR Mix (Thermo Scientific) and 250 nm of gene-specific forward and reverse primers. Each experimental set consisted of duplicate samples, non-template controls and serial dilutions of standard templates. PCR was performed in Rotor-Gene 6000 apparatus of Corbett with the following thermal cycle conditions: 15 min enzyme activation at 95°C and subsequent 40 three-step cycles of 95°C for 15 sec, 55°C for 30 sec and 72°C for 30 sec. As a reference, glyceraldehyde-3-phosphate dehydrogenase (GAPDH) and microtubule associated protein 2 (MAP2) cDNA levels were used. The rat specific primers used were the following:

GAPDH - fw 5′ TCAACGGCACAGTCAAGGC 3′, rv 5′ CGCTCCTGGAAGATGGTGAT 3′;
NSE - fw 5′ GCCGGCCTTTAATGTGATCAA 3′, rv 5′ CATGGCCAACTTGTTCCCAG 3′;
TNF - fw 5′ GAGGTCAACCTGCCCAAGTACT 3′, rv 5′ CTGGGTAGAGAACGGATGAACAC 3′;
tPA - fw 5′ TGCCCTAAGGGACCAACTGA 3′, rv 5′ TGGGTGCCACGGTAAGTCA 3′;

Emission data were analyzed by the Rotor Gene program and cycle threshold (CT) number was calculated automatically by it. We also verified that there was no detectable amplification in non-template controls. CT numbers showed a significant linear reverse correlation with logarithmic concentrations of standard templates. The original copy numbers of the first strand cDNA in RT samples were calculated from the CT numbers by fitting to the obtained standard correlation.

### Statistics

Every experiment was repeated at least 3 times, with several wells/35 mm dishes used for each treatment. The morphological data were summarized and analyzed automatically using Image-Pro Plus software. The stained neurons were counted in several fields of view in each glass. The statistical analysis of the data was done using t-tests or analysis of variance (ANOVA), after its normal distribution was verified using Shapiro-Wilk normality test. Significance was set at P<0.05.
